# Un-ruptured Non-Coronary Sinus of Valsalva Aneurysm with Complete Heart Block

**DOI:** 10.12669/pjms.336.13285

**Published:** 2017

**Authors:** Saba Hussain, Faisal Qadir, Pir Sheeraz, M. Asad Bilal Awan

**Affiliations:** 1 Dr. Saba Hussain, FCPS. Department of Cardiology, National Institute of Cardiovascular disease, Karachi, Pakistan; 2 Dr. Faisal Qadir, FCPS. Department of Cardiology, National Institute of Cardiovascular disease, Karachi, Pakistan; 3 Dr. Pir Sheeraz, MBBS. Department of Cardiology, National Institute of Cardiovascular disease, Karachi, Pakistan; 4 Dr. M. Asad Bilal Awan, FCPS. Department of Cardiology, National Institute of Cardiovascular disease, Karachi, Pakistan

**Keywords:** Sinus of Valsalva, Aortic aneurysm, Heart block, Echocardiography

## Abstract

Sinus of Valsalva aneurysm is a rare cardiac anomaly with wide spectrum of clinical presentation ranging from asymptomatic state to dreadful complications due to compression of vital surrounding structures or aortic dissection. There are only few reported cases of sinus of Valsalva aneurysm presented with cardiac arrhythmias including complete heart block. We herein, present a case of a 50 year male who presented with complete heart block. A large noncoronary sinus of Valsalva aneurysm compressing the surrounding atrioventricular junctional tissue was detected incidentally during echocardiographic study, which was considered to be the cause of patient’s complete heart block. This case report implies the importance of clinical suspicion of secondary causes like sinus of Valsalva aneurysm in patients with complete heart block and utility of echocardiography in the evaluation of heart block patients.

## INTRODUCTION

Sinus of Valsalva aneurysm (SVA) is rare cardiac anomaly usually congenital while only a minority are acquired. An unruptured sinus of Valsalva aneurysm is unique as it can present in an asymptomatic state to life threatening complications.^1^ Complete heart block is an atypical presentation of SVA caused by either compression effects or infiltration of blood and inflammatory cells into main conducting system. Literature sites few cases of SVA presenting with complete heart block.^2^-^4^ Echocardiography is a quick non invasive widely used modality for the evaluation of SVA with the accuracy of 90%.^5^

We report a middle aged patient who presented with symptomatic complete heart block, in whom a large non coronary sinus of valsalva aneurysm was detected incidentally during routine echocardiography and was considered the unusual cause of heart block in that patient.

## CASE REPORT

A 50 year male presented at the cardiac emergency department with continuous, low intensity, non radiating dull chest pain and frequent episodes of dizziness for two weeks. There was no associated history of fever, preceding infection, unconsciousness, new drug use, alcoholism or recent heavy weight lifting. Past medical history was significant for untreated hypertension and 20 pack-years cigarette smoking. There was no history of prior cardiovascular disease, thyroid disease or diabetes. Family history was negative for any known cardiac illness or sudden death. The patient was daily wage manual labourer by profession.

The patient was an average height and built male, conscious, oriented and in no apparent distress. He had blood pressure of 140/60 in both arms and a regular radial pulse at 40 beats per minute. Cardiac auscultation yielded variable S1 without any obvious murmur. Rest of physical examination was unremarkable. ECG done in emergency department revealed complete heart block with ventricular escape rhythm of 40 bpm and non specific ST-T wave changes. Patient was admitted and a temporary pace maker was inserted.

His complete blood count showed platelets count of 143x10^9^ /L, while rest of the blood workup including renal function test, liver function test, thyroid function test, serum electrolytes and 2 sets of cardiac troponin-I levels were all within normal limits. Viral serology was reactive for hepatitis C. Chest radiograph was also unremarkable.

**Fig.1:**
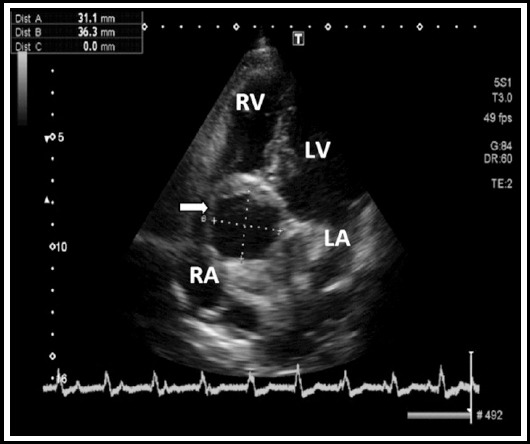
Transthoracic echo apical 5 chamber view showing non-coronary sinus aortic aneurysm (RV = Right Ventricle, LV = Left Ventricle, RA = Right Atrium, LA = Left Atrium).

A routine transthoracic echocardiogram showed normal biventricular function; however a well defined echo free space was seen along non-coronary sinus of Valsalva, protruded into the right atrium. Other pertinent findings were trileaflet aortic valve with trivial aortic regurgitation, a properly placed pace maker lead and absence of aortic dissection flap (Fig.1).

**Fig.2:**
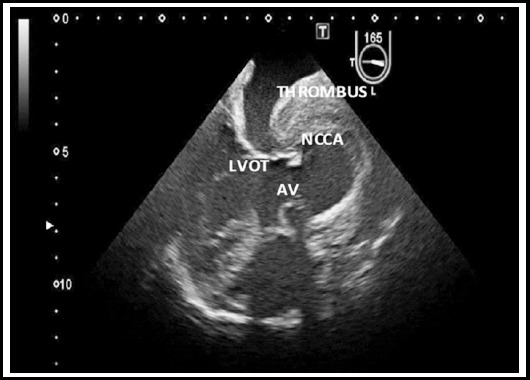
Transesophageal Echocardiography long axis view of aorta showing non-coronary cusp sinus of Valsalva aneurysm [NCCA] and thrombus (AV = Aortic Valve, LVOT = Left Ventricle Out flow Tract).

For further evaluation a Transesophgeal Echocardiography was performed next day that revealed a 5x5 cm aneurysm of noncoronary sinus with layered thrombus along its walls. Rest of aortic examination was normal and no intimal flap or shunt was detected. (Fig.2)

**Fig.3:**
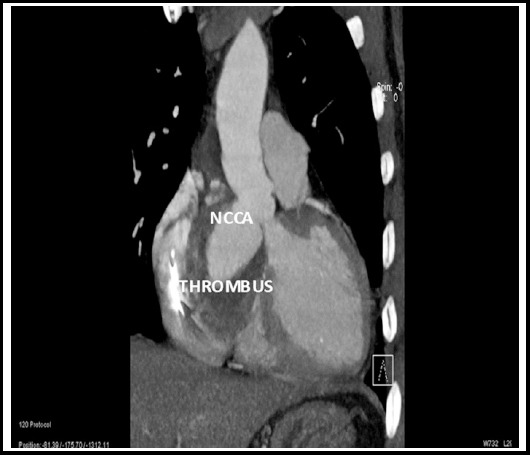
Contrast enhanced cardiac CT scan coronal view showing non-coronary cusp sinus of Valsalva aneurysm (NCCA) and thrombus.

Contrast cardiac CT scan confirmed the echocardiographic findings of noncoronary sinus of Valsalva aneurysm in close proximity of membranous ventricular septum with normal remaining aortic dimensions and no atherosclerotic changes or dissection flap. Coronary anatomy could not be adequately visualized because of respiratory artefacts. (Fig.3)

The patient remained in complete heart block and on temporary pacing. Surgical consultation was taken for the repair and excision of the mass. Coronary angiography was performed on the day of surgery showing normal epicardial coronary arteries. Aortogram was not performed for fear of dissection or thrombus embolisation.

Per-operative finding was a large non coronary sinus aneurysm filled with thrombus extending almost upto inferior vena cava. Thrombectomy and plication of aortic root aneurysm with pericardial patch placement was performed successfully. Post-operative period was complicated by hemodynamic instability and acute renal shut down. The patient succumbed to shock state and multiorgan failure and expired on third post operative day.

## DISCUSSION

Sinus of valsalva aneurysm (SVA) is an uncommon cardiac anomaly with an incidence of 0.09% in general population and 0.1-3.5% of all congenital heart defects.^1^,^6^ Approximately 65-85% SVAs originate from the right sinus of valsalva, 10-30% from noncoronary and <5% from left coronary sinus.^1^ It is usually congenital however rare cases of acquired SVAs are caused by atherosclerosis, trauma, cystic medial necrosis, syphilis and infective endocarditis. Unruptured sinus of Valsalva aneurysm’s clinical presentation varies from a typical asymptomatic state, often detected serendipitously by medical imaging performed for other purpose to life threatening conditions like myocardial infarction, heart blocks, right ventricular obstruction, infective endocarditis, tamponade or cerebrovascular embolisation or progression to aortic dissection or rupture. Transthoracic echocardiography is a quick, noninvasive and the most commonly used diagnostic tool for SVA.^7^ It provides additional information about aortic regurgitation, left ventricular dysfunction and associated abnormalities or complications.

Our patient had presented with complete heart block and sinus of Valsalva aneurysm was detected incidentally during routine echocardiography which was considered to be the causative factor for the atrioventricular block in absence of other risk factors. Unfortunately our patient did not survive after a successful surgery; for it would have been interesting to see the reversion of the heart block to normal cardiac rhythm after decompression of the atrioventricular nodal area.

Sinus of Valsalva aneurysm although rare but should be considered in the differential diagnosis of complete heart block and the readily available tool like echocardiography should always be performed before proceeding with a permanent pace maker implantation as it can sometimes provide useful information regarding somewhat rare structural causes of heart block and guide towards a different management strategy in this group of patients.

### Authors’ Contribution

***Dr Saba Hussain, Dr Pir Sheeraz and Dr Asad Bilal Awan*** collected data and were involved in manuscript writing.

***Dr Faisal Qadir*** did the manuscript review and final approval of version to be published.
